# Sehnsucht nach Veränderung – Bessere Gesundheitschancen für vulnerable Gruppen

**DOI:** 10.1007/s00103-023-03740-1

**Published:** 2023-06-29

**Authors:** Anke-Christine Saß

**Affiliations:** Abteilung für Epidemiologie und Gesundheitsmonitoring, Robert Koch-Institut, General-Pape-Str. 62–66, 12101 Berlin, Deutschland

Nach drei Jahren zum Teil gravierender pandemiebedingter Einschränkungen in allen Lebensbereichen ist nun im Sommer 2023 fast alles wieder möglich. Die Auswirkungen der Pandemiejahre sind jedoch zu spüren und die langfristigen Folgen noch nicht abschätzbar. Das betrifft sowohl die individuelle Ebene, z. B. die gesundheitlichen Langzeitfolgen einer SARS-CoV-2-Infektion, familiäre Herausforderungen oder berufliche Veränderungen, wie auch die gesamte Gesellschaft. Und selbst wenn die persönliche Bilanz nach drei Jahren „Ausnahmezustand“ für jede und jeden anders ausfällt, sind die Lasten dieser Zeit nicht zufällig oder gar gleichmäßig verteilt. In Politik und Gesellschaft ist inzwischen längst angekommen, dass es die vulnerablen, die besonders verletzlichen Bevölkerungsgruppen sind, die in der Pandemie besonders schwere Lasten tragen mussten – gesundheitlich, sozial, finanziell. Die rege wissenschaftliche Beschäftigung mit diesem Thema zeigt, wie stark auch das Bedürfnis engagierter Wissenschaftlerinnen und Wissenschaftler ist, diese Ungleichheiten zu erforschen, sichtbar zu machen und in den öffentlichen Diskurs einzubringen. Dazu soll die vorliegende Ausgabe beitragen.

Es ist die zweite von zwei Schwerpunktausgaben des *Bundesgesundheitsblattes*, die sich den Auswirkungen der COVID-19-Pandemie in den besonders vulnerablen Bevölkerungsgruppen widmen. Was bedeutet Vulnerabilität in diesem Zusammenhang? Es geht um Personengruppen, die überdurchschnittlich hohe Infektionsraten verzeichneten oder vermehrt von schweren Krankheitsverläufen betroffen waren. Für bestimmte Gruppen war die Nicht-Inanspruchnahme von Versorgungsangeboten in der Pandemie besonders problematisch. Teile der Bevölkerung wurden aufgrund ihrer Lebensumstände von den Eindämmungsmaßnahmen härter getroffen oder sie wurden von Präventionsbotschaften und Impfangeboten schlechter erreicht.

Sehr unterschiedliche Personengruppen haben sich in der Pandemie als vulnerabel erwiesen. In der ersten Ausgabe, das im März 2023 erschienen ist, wurde der Fokus auf Vulnerabilität aufgrund physischer Risiken durch Alter, chronische Krankheit und Behinderung gelegt. Der letzte Themenblock der Ausgabe widmete sich den vulnerablen Lebensphasen Schwangerschaft und Geburt. Die nun vorliegende zweite Ausgabe richtet den Blick auf Vulnerabilität aufgrund der sozialen Lage. Untersucht werden u. a. der Einfluss der Wohn- und Arbeitsbedingungen auf die Gesundheit in der Pandemie und besondere Risiken, die durch eine Migrationserfahrung entstehen können.

Der erste Themenblock „Gesundheit von sozial schlechter gestellten Menschen in der Pandemie“ der vorliegenden Ausgabe enthält drei Beiträge, in denen demografische und sozioökonomische Merkmale als Einflussfaktoren für unterschiedliche Gesundheitsoutcomes beschrieben werden. *Finne und Razum* analysieren Daten des Sozio-oekonomischen Panels (SOEP) aus den Jahren 2018–2020 und nehmen die Personen in den Blick, deren subjektives Wohlbefinden im ersten Jahr der COVID-19-Pandemie besonders beeinträchtigt war. Sie finden keine klar abgrenzbaren Risikogruppen für Einbußen im Wohlbefinden. Der Gesundheitszustand vor Pandemiebeginn scheint bedeutsamer zu sein als soziodemografische und -ökonomische Merkmale. *Reibling et al.* untersuchen empirisch den Zusammenhang zwischen COVID-19-bezogener Selbststigmatisierung im Winter 2020/2021 und Vulnerabilität. Sie finden höhere Werte für Selbststigmatisierung (z. B. sich schuldig fühlen bei einer Infektion; Angst, jemandem davon zu erzählen) bei Personen mit gesundheitlicher Vulnerabilität (z. B. schlechter Gesundheitszustand, Vorerkrankungen). Auch in dieser Studie spielen soziodemografische Variablen eine geringere Rolle, bis auf Geschlecht: Bei Frauen tritt Selbststigmatisierung häufiger auf als bei Männern. Ein deutlicher Einfluss soziodemografischer Merkmale zeigt sich hingegen beim COVID-19-Impfstatus. Das berichten *Haß et al*. in ihrem Beitrag mit Ergebnissen aus der Begleitforschung zur Kommunikation der Corona-Schutzimpfung in Deutschland (CoSiD-Studie). Die Impfquote steigt tendenziell mit dem Alter, dem Bildungsgrad sowie dem Haushaltseinkommen und ist höher unter Personen in den alten Bundesländern und ohne Migrationshintergrund. Mit Blick auf das Informationsverhalten zeigt sich, dass der Anteil von Menschen ohne Impfabsicht unter anderem bei denjenigen höher war, die sich in den sozialen Medien über die Corona-Schutzimpfung informiert hatten.

Im nächsten Themenblock geht es um die Bedeutung der Arbeitswelt für die Gesundheit in der Pandemie. Krankenkassendaten belegen eindrücklich, dass das Risiko, an COVID-19 zu erkranken, schwer zu erkranken oder zu versterben, in bestimmten Berufen erhöht ist. *Wahrendorf et al. *analysieren die Daten von mehr als drei Mio. Versicherten und finden erhöhte Erkrankungsrisiken insbesondere in Gesundheitsberufen. Zudem sind die Risiken für Krankenhausausaufenthalte und Mortalität bei Beschäftigten in Reinigungsberufen, Verkehrs- und Logistikberufen erhöht. Für alle drei Outcomes gilt: Personen in Berufen ohne Leitungsfunktion und mit geringem Anforderungsniveau sind stärker gefährdet (höchstes Risiko für Helfertätigkeiten). Die selbst eingeschätzte Gesundheit von Personen, die aufgrund ihres geringen Einkommens trotz Erwerbsarbeit armutsgefährdet sind, wird im Beitrag von *Pförtner und Demirer* betrachtet. Sie nehmen einen Beobachtungszeitraum von 27 Jahren in den Blick und schlussfolgern, 1) dass sich die subjektive Gesundheit im Zuge der COVID-19-Pandemie verbessert hat (betrifft alle erwerbstätigen Studienteilnehmenden), 2) dass die Unterschiede in der Gesundheit zuungunsten Erwerbsarmer persistieren. Insbesondere Personen, die im Erwerbsleben häufiger von Erwerbsarmut betroffen waren, sind gesundheitlich gefährdet.

Die Bedeutung der Wohnsituation für die Gesundheit in der COVID-19-Pandemie wird im Beitrag von *van Rüth et al.* adressiert. Die Autorinnen und Autoren berichten auf Basis eines Literaturreviews und einer eigenen Studie von der Situation wohnungsloser Menschen in der Pandemie. Sie sind häufig psychisch und somatisch erkrankt und haben einen eingeschränkten Zugang zum medizinischen Regelsystem. Versorgungseinrichtungen mit Gruppenräumen und Schlafsälen erhöhen das Risiko für Ausbruchsgeschehen. Vermutlich gab es eine hohe Rate an unwissentlich infizierten wohnungslosen Menschen. Trotzdem war ein unkontrolliertes COVID-19-Ausbruchsgeschehen, vor dem einige Wissenschaftlerinnen und Wissenschaftler zu Beginn der Pandemie gewarnt hatten, nicht zu beobachten.

Der nächste Themenblock widmet sich der Gesundheit von Menschen mit Migrationsgeschichte in der Pandemie. Im ersten Beitrag von *Gold et al.* wird noch einmal das Thema Wohnen aufgegriffen. Geflüchtete Menschen in Sammelunterkünften sind durch eine hohe Belegungsdichte und gemeinschaftlich genutzte Räume einem erhöhten SARS-CoV-2-Infektionsrisiko ausgesetzt. Die Pandemiebewältigung (z. B. Aufklärung, Testung, Quarantäne) durch die Verantwortlichen vor Ort wurde in Interviews erfasst. Dabei lag der Schwerpunkt auf der Zusammenarbeit der Aufnahmebehörden. Die Autorinnen und Autoren leiten Empfehlungen für eine zukünftig verbesserte Krisenreaktion ab. Die Lebenszufriedenheit von Menschen mit Migrationsgeschichte wird in einer empirischen Arbeit von *Koschollek et al.* untersucht. Sie war bei Teilnehmenden mit erwartetem bzw. bereits eingetretenem Arbeitsplatz- und Einkommensverlust (in den Jahren 2021/2022) geringer. Die Autorinnen und Autoren weisen auf die sozial ungleiche Verteilung solcher indirekter sozioökonomischer Pandemiefolgen hin: Menschen mit Migrationsgeschichte sind häufiger betroffen. Das hat Folgen für die Gesundheit.

Im vorletzten Beitrag der Ausgabe geht es um besondere Belastungen von Familien in der Corona-Pandemie. Die Auswirkungen von „Homeschooling“ für Kinder und Familien sind inzwischen gut untersucht und Nachteile empirisch belegt. Dies gilt insbesondere für von Armut betroffene Familien. Die Situation von Familien mit kleinen Kindern wird in der von *Renner et al.* präsentierten Studie untersucht. Hier zeigt sich, dass auch Eltern mit Kindern im Alter von 0 bis 3 Jahren ihre Situation in der COVID-19-Pandemie als belastend erlebt haben. Die soziale und affektive Entwicklung der Kinder wurde durch die Pandemie negativ beeinflusst, das bestätigen kinderärztliche Untersuchungsbefunde, die ergänzend ausgewertet wurden. Die Effekte sind bei Kindern aus armutsbelasteten Familien stärker ausgeprägt.

Die Auswirkungen traumatischer Erfahrungen auf die psychische Gesundheit während der COVID-19-Pandemie sind Thema des letzten Beitrags, hierzu zählen auch Erlebnisse häuslicher Gewalt vor oder während der Pandemie. Zentrale Forschungsbefunde aus dem deutschsprachigen Raum wurden von *Lotzin et al.* zusammengetragen. Es zeigt sich, dass Erwachsene mit vorbestehenden oder aktuellen traumatischen Erfahrungen während der Pandemie eine erhöhte psychische Belastung im Vergleich zu Erwachsenen ohne solche Erfahrungen aufwiesen. Eine Reihe von Risikofaktoren (z. B. weibliches Geschlecht, geringe Sozialkontakte) erhöhte das Risiko für psychische Belastungen während der Pandemie. Traumabetroffene stellen eine besonders vulnerable Gruppe dar, die spezifische Unterstützungsbedarfe aufweist.

Die wissenschaftliche Beschäftigung mit den Auswirkungen der unmittelbaren Eindämmungsmaßnahmen und Pandemiefolgen auf besonders verletzliche Bevölkerungsgruppen ist in vollem Gange. Die Ergebnisse sind vielfältig, nicht alle Mechanismen sind schon verstanden. Die langfristigen Auswirkungen der Pandemie sind Gegenstand weiterer, noch laufender Forschungsprojekte. Neben der Generierung von Evidenz für Zusammenhänge besteht die Aufgabe nun vor allem darin, die Erkenntnisse zu teilen und Akteurinnen und Akteure zu erreichen, die sich für Veränderungen einsetzen. „Sehnsucht nach Veränderung“, mit diesem Titel erschien 1989 eine Langspielplatte (LP) mit Instrumentalmusik der Band „L’art de Passage“ – Musette aus Frankreich, Blues aus Louisiana, Tango aus Argentinien. In der Musik drückte sich das Lebensgefühl vieler Menschen in der DDR aus. Die Sehnsucht nach Veränderung mündete in großen gesellschaftlichen Umbrüchen, deren Spuren bis in die heutige Zeit reichen. Was können wir dafür tun, dass die Lessons Learned der Pandemie in notwendigen Veränderungen münden? Wer muss informiert werden, wer muss überzeugt werden? Gelingt es uns, die Pandemie als Impuls zu nutzen, um gesundheitliche Chancengleichheit für alle Bevölkerungsgruppen zu fördern? Ein gleichwertiger Zugang zu gesunden Lebensweisen und medizinischer Versorgung für alle Menschen bleibt ein vorrangiges Public-Health-Ziel.

Ein herzlicher Dank gebührt Frau Dr. Anke Spura von der Bundeszentrale für gesundheitliche Aufklärung (BZgA) für ihre Impulse bei der Konzeption dieser Ausgabe.

